# A Robust Framework for Epidemic Analysis, Prediction and Detection of COVID-19

**DOI:** 10.3389/fpubh.2022.805086

**Published:** 2022-05-06

**Authors:** Farman Hassan, Saleh Albahli, Ali Javed, Aun Irtaza

**Affiliations:** ^1^Department of Computer Science, University of Engineering and Technology, Taxila, Pakistan; ^2^Department of Information Technology, College of Computer, Qassim University, Buraydah, Saudi Arabia; ^3^Department of Computer Science and Engineering, Oakland University, Detroit, MI, United States; ^4^Department of Computer and Electrical Engineering, University of Michigan, Dearborn, MI, United States

**Keywords:** prediction, Coronavirus - COVID-19, Time-series (TS) model, gaussian, mathematical model, epidemic spreading algorithm

## Abstract

Covid-19 has become a pandemic that affects lots of individuals daily, worldwide, and, particularly, the widespread disruption in numerous countries, namely, the US, Italy, India, Saudi Arabia. The timely detection of this infectious disease is mandatory to prevent the quick spread globally and locally. Moreover, the timely detection of COVID-19 in the coming time is significant to well cope with the disease control by Governments. The common symptoms of COVID are fever as well as dry cough, which is similar to the normal flu. The disease is devastating and spreads quickly, which affects individuals of all ages, particularly, aged people and those with feeble immune systems. There is a standard method employed to detect the COVID, namely, the real-time polymerase chain reaction (RT-PCR) test. But this method has shortcomings, i.e., it takes a long time and generates maximum false-positive cases. Consequently, we necessitate to propose a robust framework for the detection as well as for the estimation of COVID cases globally. To achieve the above goals, we proposed a novel technique to analyze, predict, and detect the COVID-19 infection. We made dependable estimates on significant pandemic parameters and made predictions of infection as well as potential washout time frames for numerous countries globally. We used a publicly available dataset composed by Johns Hopkins Center for estimation, analysis, and predictions of COVID cases during the time period of 21 April 2020 to 27 June 2020. We employed a simple circulation for fast as well as simple estimates of the COVID model and estimated the parameters of the Gaussian curve, utilizing a parameter, namely, the least-square parameter curve fitting for numerous countries in distinct areas. Forecasts of COVID depend upon the potential results of Gaussian time evolution with a central limit theorem of data the Covid prediction to be justified. For gaussian distribution, the parameters, namely, extreme time and thickness are regulated using a statistical *Y*^2^ fit for the aim of doubling times after 21 April 2020. Moreover, for the detection of COVID-19, we also proposed a novel technique, employing the two features, namely, Histogram of Oriented Gradients and Scale Invariant Feature Transform. We also designed a CNN-based architecture named COVIDDetectorNet for classification purposes. We fed the extracted features into the proposed COVIDDetectorNet to detect COVID-19, viral pneumonia, and other lung infections. Our method obtained an accuracy of 96.51, 92.62, and 86.53% for two, three, and four classes, respectively. Experimental outcomes illustrate that our method is reliable to be employed for the forecast and detection of COVID-19 disease.

## Introduction

In March 2020, the World Health Organization (WHO) confirmed a widespread of a novel Corona Virus called COVID-19, a pandemic. COVID-19 is caused by a virus named severe acute respiratory syndrome coronavirus 2 (SARS Cov2). Initially, the pandemic started in Wuhan, China; however, it spread quickly to a large part of the globe (1). COVID-19 spreads *via* breathing drops of the diseased individual, which are generated when the infected person sneezes or coughs. The droplets of an infected person can also contaminate large surfaces that increase the spread more quickly. The infected person may suffer respiratory illness either severe or mild; however, the severe may need the support of ventilation (2). People of old age and those having chronological illnesses are prone to COVID-19 infection. Therefore, many countries shut their international borders and imposed strict presentation measures to avoid a quick spread of the COVID-19 (3).

Researchers and scientists have developed different vaccines to combat the pandemic by sequencing ribonucleic acid (RNA) from COVID-19. The organizations of vaccines employed both conventional and leading-edge technology with six different platforms of vaccine, such as deoxyribonucleic acid (DNA), messenger RNA (mRNA), viral vector-based, subunit or protein, inactivated virus, and a live attenuated virus. However, the developed vaccines can significantly reduce the quick spread and enhance immunity by producing antibodies. The vaccines have shown 95% effectiveness; however, some issues were encountered while managing the vaccines, i.e., the hesitancy of vaccine, complacency, and logistical challenges of the supply chain. Most importantly, the vaccines are not to cure rather a prevention measure against COVID-19 (4). Although, vaccines are produced, however, detection is crucial as it assists in easily tracking the persons who were in touch with the infected person. The quick spread of the pandemic is significantly avoidable by tracing these people. In the initial stage, the infection manifests as an infection of the lungs; hence, the researchers utilized the lung's x-rays and computed tomography (CT) images to detect the lungs infection (5).

Numerous models have been designed to predict the infectious disease that quickly spread similar to the COVID-19. Recently, a model named susceptible-infected-removed (SIR) (6, 7) has been employed for estimating the spread and fatality rate of COVID. Distinct variations of these systems are either very simple so that they cannot accurately generate the predictions, or either very complex for understanding. The early forecasting of certain attributes for COVID-19, namely, the highest quantity of positive cases, the fatality rate per day, forecasting peak number, the exact time of new severe sick people per day (SSPs), is believed to be significant for each country, especially those that are expected to witness exponential growth. More specifically, the quick and dependable forecasting of COVID is significant for the policymakers to enhance the monitoring of pandemic drift and to take precautionary measures for avoiding the shortage of life-saving resources in medical centers as well as in emergency services. In this work, we present the development as well as utilization of the Gaussian model as a beneficial, simple, and effective description of fatalities due to COVID-19 with time and the recent works in the USA (8) and Germany (9). Distinct from the prior study, we chose to use a knowledgeable regular death rates algorithm (10) as evaluated input data. Moreover, we also presented the Gaussian doubling times principle as an amount of an increased rate (11) as an alternative to the growing infections. The Gaussian distribution function assessment has a significant role to resolve various problems in plasma kinetic theory named drift-Maxwellian (12) or counter streaming bi-Maxwellian (13) velocity distribution function. The above-mentioned terms are called plasma physics.

Accurate and timely detection of COVID-19 is important for controlling the quick spread of this disease among people. It has become more crucial to detect the COVID-19-infected people after the vaccination to quarantine the people and to prevent the spread. The PT-PCR is believed to be a standard detection method for COVID; however, PT-PCR generates a lot of false positives due to various reasons, namely, stages of the disease, technique of collecting specimens, disadvantages of methodology that sustainably delay the control and detection process. The sensitivity and specificity of the initial standard testing method have been dejected in these works (14–17). Hence, we required a unique automatic diagnostic method, which can assist to stop the quick spread of COVID-19 (18).

Medical experts, clinicians, technologists, and researchers are putting their efforts to early detect the patients with COVID-19. In 2019, more than 755 research articles were published as reported by PubMed (19), while, in the first 3 months of 2020, more than 1,245 articles were published. Deep learning (DL) and artificial intelligence methods are utilized by scientists for the detection of COVID-19 using CT and chest x-rays images (CXI). DL techniques (20–26) have shown extraordinary results in research applications and are commonly employed due to the enhanced performance comparative to the conventional techniques. Compared to machine learning and conventional techniques, features are not selected manually. On the other hand, the DL model can be trained by changing the configurations and parameters to learn the prominent features from the dataset. The research community has examined the DL techniques to explore the medical imaging field before the COVID-19 pandemic. DL attained maximum attention to detect COVID-19 using CXI. Researchers reported detailed methods (27, 28) to detect COVID-19 through computer vision and artificial intelligence.

For many papers, transfer learning-based techniques are the go-to methods. In transfer learning, the pre-trained models on the ImageNet dataset are employed for performing the transfer learning. Even though methods are the same, however, distinct architectures are employed in works (29). Distinct variants are employed even if the architectures are the same. Cross-validation is also considered in transfer learning. Additionally, techniques with novel CNN models are also employed that use the significance of transfer learning when the available data are small for training. In (30), a CNN-based architecture named COVID-Net was designed for the detection of COVID-infected patients through CXI. Authors also introduced a dataset COVIDx that has three classes, i.e., normal, COVID, and viral pneumonia (VP). COVID-Net is based on the two phases of projections, such as depth-wise representation, expansion, and extension. Initially, the CNN was trained on ImageNet as well as on the COVIDx dataset. In (31), a model that comprises three portions, such as a backbone, a classification head, and an anomaly detection head, was developed for the detection of COVID-infected people. The backbone part was used on ImageNet for extracting the high-level feature from CXI, and the extracted features were passed into other two parts of the network such as classification and anomaly heads to generate a score. A cumulative score of “one” was also used for every prediction. In (32), a capsule network-based model named COVID-CAPS was designed for the detection of COVID-19 through CT scans and CXI. It was reported that the benefit of employing a capsule network is it performs good, while the training data are small. The COVID-CAPS was trained using the dataset (33). In (34), a CNN-based model, namely, DeTraC was developed that comprises three stages, such as feature extraction, decomposition, and the third stage, a class composition. The backbone architecture was employed to obtain features from images, followed by using SGD optimizer and, finally, a class to categorize images into normal or COVID-19 infected. In (35), COVIDLite was developed that employed a depth-wise separable CNN to classify the CXI for the detection of COVID-19. Similarly, this (36) also employed depth-wise separable convolutional layers in the XceptionNet architecture (37) and named it a Fast COVID-19 detector. To improve the color fidelity, white balancing was used, while, to expand the visibility and optimize the white balance, preprocessing was executed. In (38), the CNN model was designed that comprises a block of convolutional layers, having 16 filters, a batch normalization layer, an activation function ReLU, two fully connected layers, followed by a SoftMax layer. In (39), a set of customized CNN models was employed for the prediction of an infection graph. Additionally, viral and bacterial pneumonia were also detected using the CNN-based model. In (40), a tailored CNN was employed that takes the fused set of features by employing two models, namely, Xception and ResNet50V2. A fused set of features was fed into the convolutional and classification layer for the classification purposes. Similarly, in (41), deep features were obtained by employing the MobileNet, and the deep features are fed into the global pooling and fully connected layer. The performance of the model was evaluated by transfer learning, training from the scratch, and fine-tuning the network. The CoroNet (41) was used to classify the x-ray images into four distinct classes, such as normal, viral and bacterial pneumonia, and COVID-19. Xception was used as a base model; however, the last two layers, such as dropout and two fully connected layers, were added. In (42), DarkCovidNet was designed for COVID-19 detection, which is based on the Darknet-19 (43). DarkCOVIDNet used a smaller number of layers than Darknet-19. Two layers, such as average pooling and SoftMax, were added for classification. In (44), a four-stage technique, namely, exemplar-based pyramid feature producing, relief, iterative principal component analysis, and classification, was developed to detect patients with COVID-19. The feature extraction was emphasized by the initial three stages, while, in the last stage, a deep neural network and artificial neural network were used for classification purposes. In (45), CovXNet with depth-wise convolutional layers was developed for binary as well as a multi-class classification problem. The model was trained from the scratch as well as used numerous modifications, such as fine-tuning, transfer learning.

In this work, we addressed the challenges that are associated with predicting and detecting COVID earlier by proposing a novel framework to reliably analyze, predict, and detect COVID-19. Moreover, the proposed framework is capable of effectively detecting VP, as well as extra lung infections.

Major contributions of the proposed study are given as follows:

- We used Gaussian doubling times for best analysis in addition to the prediction of COVID-19 globally.
- We developed an innovative COVID detector, which employs two features, namely, scale invariant feature transform (SIFT) and histogram of oriented gradients (HOG).
- We developed a novel CNN-based architecture called COVIDDetectorNet to effectively detect the patients with COVID-19 and patients suffering from VP, and other infections of the lungs.
- The proposed COVID detection technique has capability to detect normal, COVID, VP, as well as other lung infections.
- To detect COVID and other lungs abnormalities, we have performed rigorous experiments on the publicly available dataset, namely, the COVID Radiography dataset.

The rest of this manuscript is structured as follows: Section 2 Materials and Methods has a detailed explanation of our proposed working mechanism to detect the COVID-19 and estimate an infection rate. Section 3 Proposed Method gives an explanation of experimental outcomes, while, finally, Section 4 Results and Analysis has concluded the work.

## Materials and Methods

This section provides an in-depth summary of data and techniques for the COVID-19 infections, forecasting in Asian countries and globally. Moreover, the detailed discussion of the proposed CNN-based architecture, i.e., COVIDDetectorNet is presented to detect COVID-infected people, VP, and other lung infections.

### Forecasting Data

For forecasting the infection rate, we collected the data through a real-time inquiry from Johns Hopkins University as well as additional suppliers, namely, WHO, to examine and make a forecast about the pandemic for worst-hit countries. Currently, the COVID data are gathered from numerous sources, such as media reports, online news, as well as official reports of governments, etc. It is significant to consider the data of all sources as it will be helpful to examine the diverse data to have a clear as well as a comprehensive image of an epidemic and its implications.

### Scientific Simulation for Forecasting

The statistics and literature (46) demonstrate that there are three stages of a pandemic, namely, the total of infected people grows exponentially, the peak of an epidemic, and the quick decline in the infectious rate (9). Therefore, we employed a Gaussian curve to illustrate the progress of a pandemic. *K*_*p*_represents the amount of COVID-affected individuals' each day *p*, which is illustrated through a Gaussian curve as follows:


(1) $$\begin{array}{r} K\left(p\right)=P_{0}e^{-{\left(\frac{P-D}{\Delta }\right)}^{2}} \end{array}$$

In the above Equation (1), *P*_0_ represents the highest amount of infectious cases each day *D*, while Δ shows a standard deviation of a curvature.

Change in the level of infection is computed through separating *K*(*p*) w.r.t *p*. Hence, change in relative rate *R*(*p*) is given the following Equation (2).


(2) $$\begin{array}{r} R\left(k\right)=\frac{\frac{dk\left(p\right)}{dp}}{k\left(p\right)}=\frac{d\ln k\left(p\right)}{dp}=\frac{2\left(D-p\right)}{\Delta^{2}} \end{array}$$

### Doubling Time Expression

The number of cases per day can be computed by Equation (3) in terms of doubling time *E*.


(3) $$\begin{array}{r} kk_{obs}\left(p\right)=k_{obs0}e^{\frac{p\ln\;2}{E}} \end{array}$$

Similarly, relative change can be computed by the Equation (4).


(4) $$\begin{array}{r} R\left(p\right)=\frac{\frac{dk_{obs}\left(p\right)}{dp}}{k_{obs}\left(p\right)}=\frac{d\ln k_{obs}\left(p\right)}{dp}=\frac{\ln\;2}{E} \end{array}$$

The doubling time in terms of D and Δ is computed by combining the equations (2) and (4) as follows;


(5) $$\begin{array}{r} C\left(D,p\right)=\frac{\ln\;2\Delta^{2}}{2\left(D-p\right)}=\frac{0.35\Delta^{2}}{D-p} \end{array}$$

when at *p* = 0,


(6) $$\begin{array}{r} C\left(D,0\right)=\frac{0.35\Delta^{2}}{D} \end{array}$$

We required calculation of doubling time so as to obtain two values, namely, *D* and Δ. It is computed, applying the Equation (4).


(7) $$\begin{array}{r} C\left(p\right)=\frac{P*\ln\;2}{\ln\frac{E\left(Y+P\right)}{E\left(Y\right)}} \end{array}$$

In the Equation (7), the *E*(*Y*) denotes the amount of COVID cases on Day *Y*, while *P* represents a rolling window. In this work, we used a rolling window of 7.

### Doubling Time for Worldwide Cases

**Figure 1:**
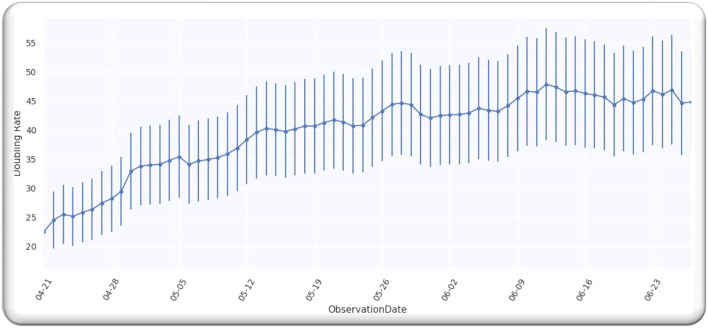
Worldwide cases doubling rate.

Figure 1 depicts doubling time for worldwide infection cases from 21 April 2020. We selected this date because the doubling rate was stabilized globally from the above-mentioned date, as every country started releasing the data publicly. Moreover, we analyzed the data till 27 June 2020 and the analyzed data assumingly it has an error of 20%. We used the data (9). In order to obtain the value of *D*, we analyzed the doubling rate at *p* = 0,


(8) $$\begin{array}{r} C\left(D,0\right)=\frac{0.35\Delta^{2}}{D}=22.6\Rightarrow 0.35\Delta^{2}=22.6D \end{array}$$

From Equations (5) and (8)


(9) $$\begin{array}{r} C\left(D,p\right)=\frac{0.35\Delta^{2}}{D-p}=\frac{22.6E}{D-p}=\frac{22.6}{1-p/D} \end{array}$$

Figure 1 illustrates a Gaussian model worldwide, doubling the rate from the date 21 April 2020 to 27 June 2020, with an error of 20%.

#### Statistical Fit for Worldwide Cases

We computed the value of *D* for the Gaussian curve of worldwide cases by computing a *Y*^2^ fit using Equation (10).


(10) $$\begin{array}{r} Y^{2}\left(D\right)=\Sigma_{l=0}^{M}{\left(\frac{n\left(p_{l}\right)-C\left(D,p_{l}\right)}{\delta \left(p_{l}\right)}\right)}^{2} \end{array}$$

The *n*(*p*_*l*_) in Equation (10) represents the analyzed doubling rate, *C*(*D, p*_*l*_) shows the estimated doubling rate, and δ(*p*_*l*_) represents an error for the analyzed rate with almost 20% and by employing the Equation (9); we got the following expression:


(11) $$\begin{array}{r} Y^{2}\left(D\right)=\Sigma_{l=0}^{M}{\left(\frac{n\left(p_{l}\right)-\frac{22.6}{1-p_{l}/D}}{.2m\left(p_{l}\right)}\right)}^{2} \end{array}$$

**Figure 2:**
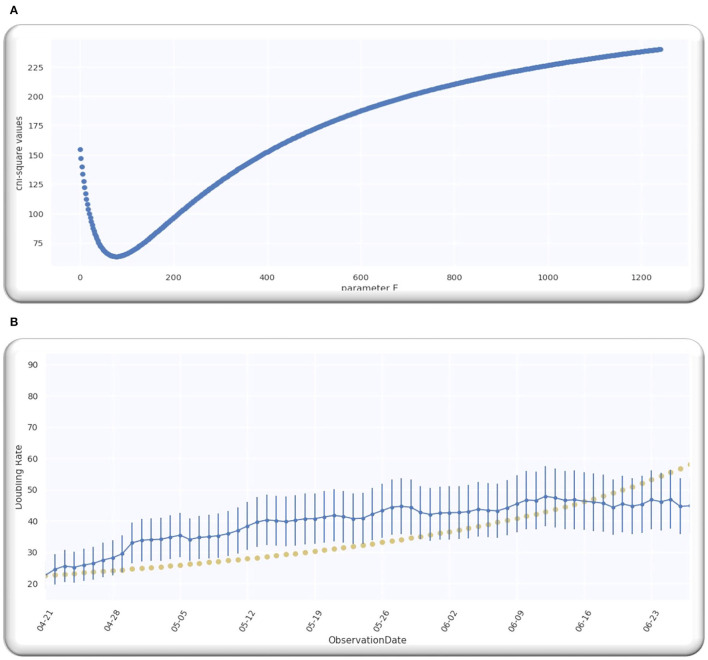
**(A)** The best estimate of E. **(B)** The doubling rate of global cases with estimates.

From the analysis till 27 June 2020, M = 67; hence, D is a single-free parameter, and the freedom degree is computed by *M*−1 = 66, while the lowest value of $Y_{\min}^{2}\;$is equal to 63.28 by using D equals to 109.5 days from *p* = 0 on 21 April 2020 as illustrated in Figure 2A.

Ratio $Y_{\min}^{2}/\left(M-1\right)$ is equal to 0.96 that signifies that model is performing well on the data because the ratio is < Value 1. Figure 2B illustrates the analyzed doubling rates with modeled doubling rates.

The value of D is equal to 109.5 when *p* = 0 on 21 April 2020, comparable to 21 April 2020 + D, which is 08/08/2020. Then, the best fit Gaussian doubling time is given by,


(12) $$\begin{array}{r} C\left(D,p\right)=\frac{22.6}{1-p/D}=\frac{22.6}{1-p/109.5}=\frac{2474.7}{109.5-p} \end{array}$$

**Figure 3:**
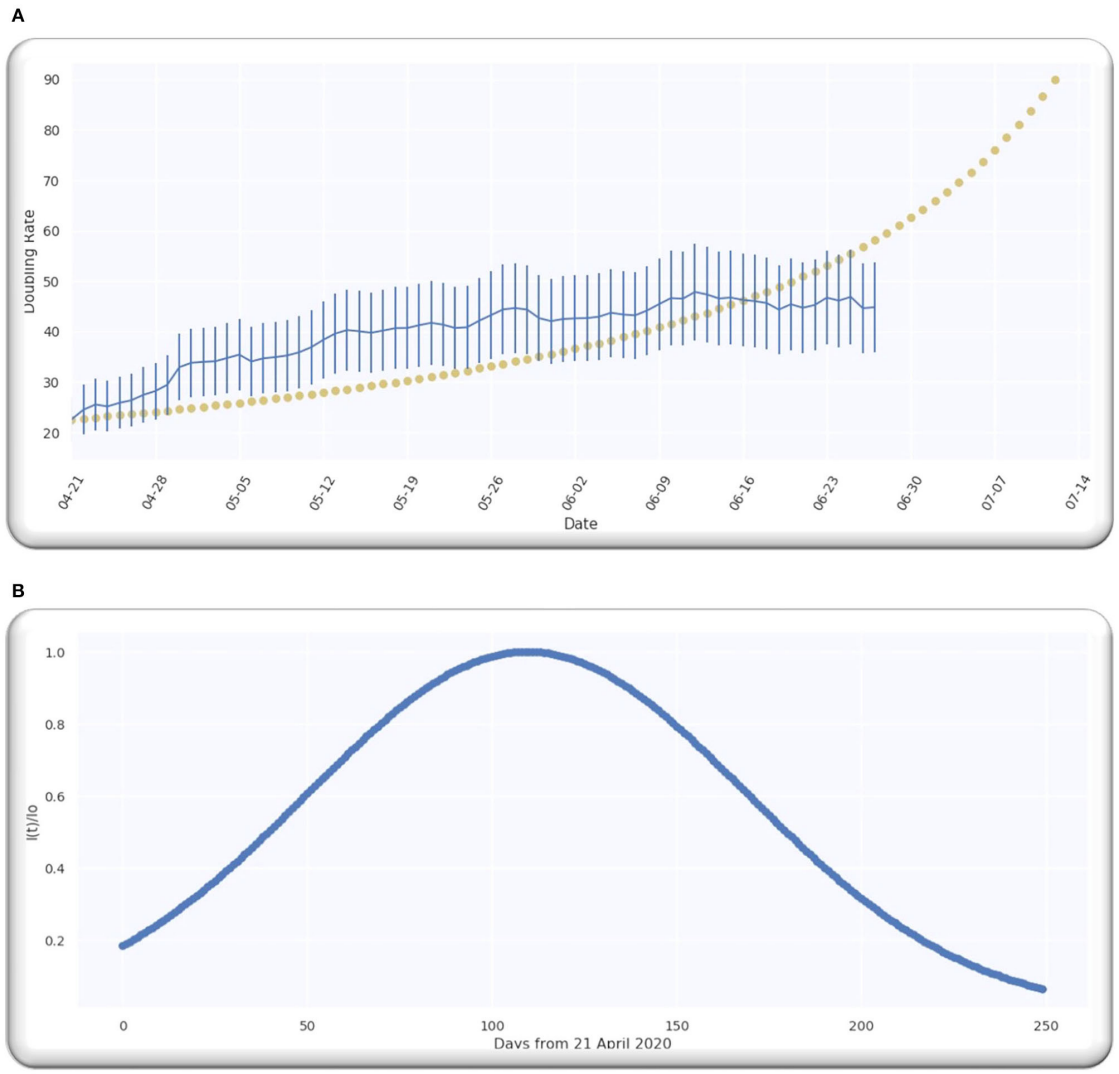
**(A)** The doubling rate of global cases with estimates. **(B)** Gaussian function for global cases.

The estimate of the doubling rate up to 14/07/2020 is illustrated in Figure 3A. We have the value of D, which is equal to 109.5, so we can compute the Δ by using Equation (8).


(13) $$\begin{array}{r} \Delta =84.09 \end{array}$$

For the worldwide infection cases, the Gaussian function in terms of the ratio of $\frac{k\left(p\right)}{k_{0}}$ is illustrated in Figure 3B.

### Doubling Time in Asia for Infection Cases

**Figure 4:**
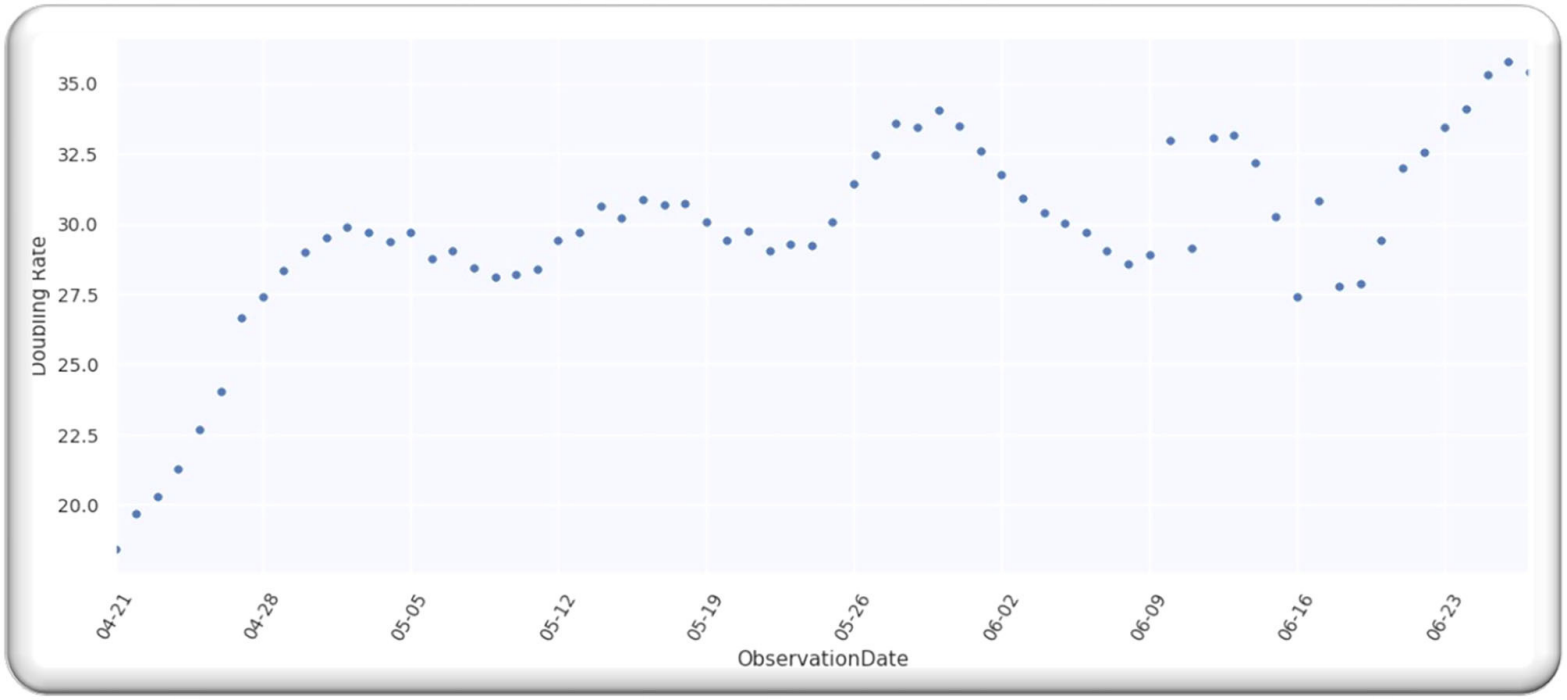
The doubling rate of cases in Asia.

The doubling time in Asia for infection cases from 21 April 2022 is illustrated in Figure 4. We have selected five different countries from Middle East, namely, Saudi Arabia, Pakistan, Turkey, Iran, and India, to make sure that all the observations are statistically related and within the same time frame from the start of the spread in various countries.

As stated before, we analyzed the data up to 27 June 2022, having an error of 20% assumption using the data (9). We examined the analyzed doubling rate for the same Asian countries that are mentioned above when the value of *p* = 0, that is


(14) $$\begin{array}{r} C\left(D,0\right)=18.42D \end{array}$$

Utilizing two Equations (5) and (14)


(15) $$\begin{array}{r} C\left(D,p\right)=\frac{18.42}{1-p/D} \end{array}$$

**Figure 5:**
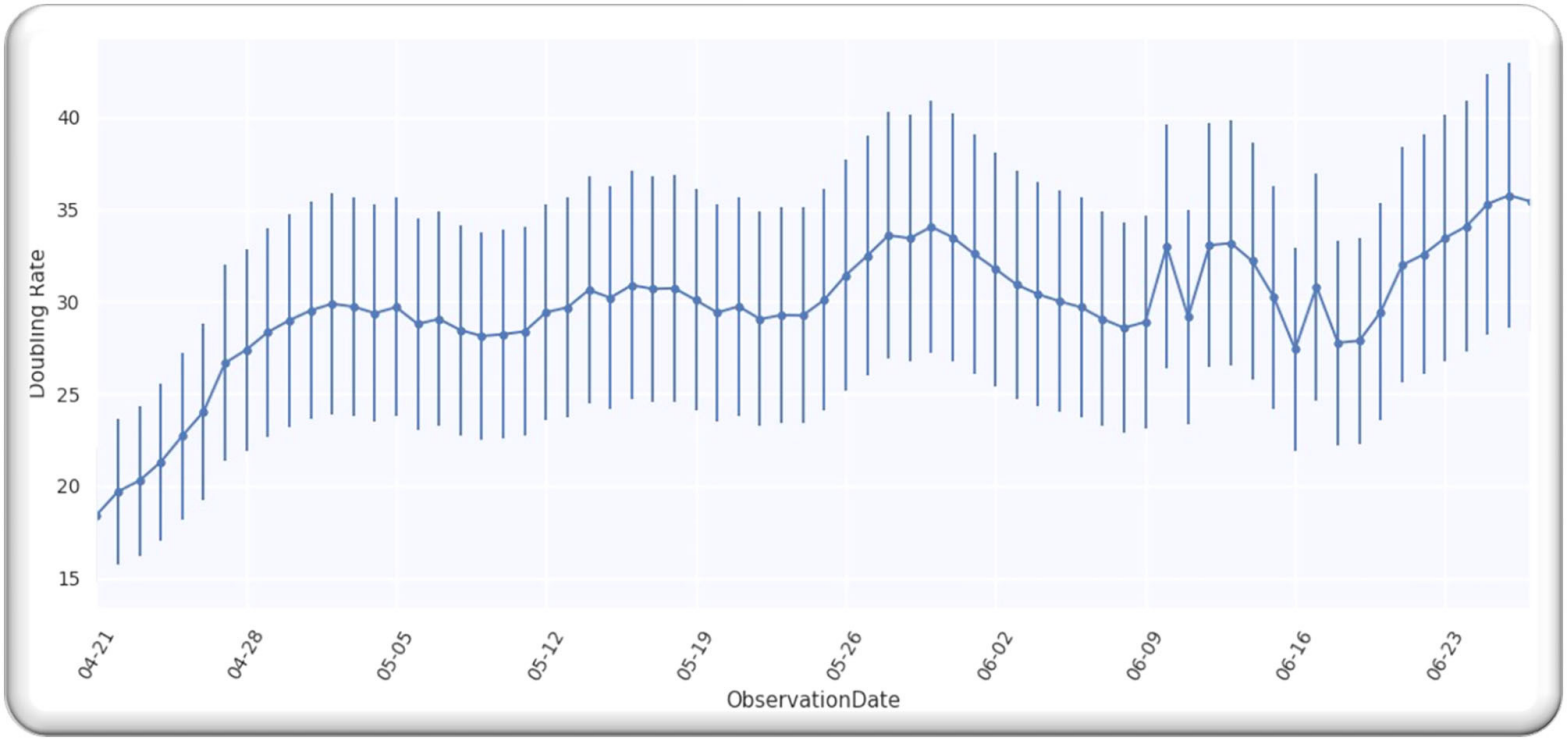
The doubling rate of cases in Asia.

Figure 5 illustrates the doubling rate, starting from 21 April 2020 to 27 June 2020, with an error of 20% for the above-mentioned Asian countries.

#### Statistical Fit for Cases in Asia

For the Gaussian curve of infection cases in Asian countries, we computed the value of D, and the Gaussian curve is determined by conducting a *Y*^2^ fit, and it is computed by using the Equation (16).


(16) $$\begin{array}{r} Y^{2}\left(D\right)=\Sigma_{l=0}^{M}{\left(\frac{n\left(p_{l}\right)-C\left(D,p_{l}\right)}{\delta \left(p_{l}\right)}\right)}^{2} \end{array}$$

In the above Equation (16), *n*(*p*_*l*_) is the analyzed doubling rate, while *C*(*D, p*_*l*_) represents an estimated doubling rate, and δ(*p*_*l*_) shows an error term for the analyzed rate, particularly for the Asian countries. The error is 20%, and, by employing the Equation (14), we got the following Equation (17):


(17) $$\begin{array}{r} Y^{2}\left(D\right)=\Sigma_{l=0}^{M}{\left(\frac{n\left(p_{l}\right)-\frac{18.42}{1-p_{l}/D}}{0.2n\left(p_{l}\right)}\right)}^{2} \end{array}$$

**Figure 6:**
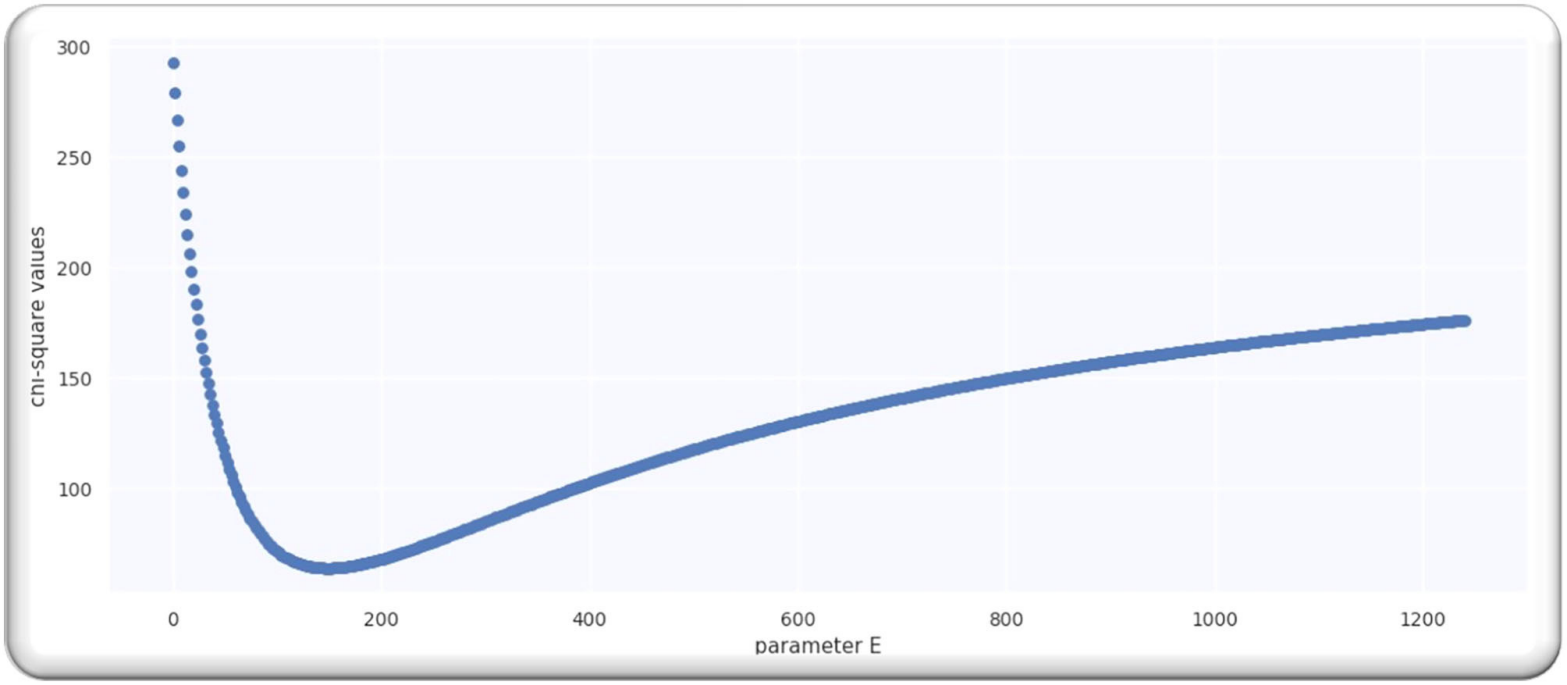
The best estimate of E.

From the analysis till 27 June 2020, M = 67; hence, D is a single-free parameter, and the freedom degree is computer by *M*−1 = 66, while the lowest value of $Y_{\min}^{2}$is equal to 64.02 by using D equals to 127 days from *p* = 0 on 21 April 2020, as illustrated in Figure 6.

**Figure 7:**
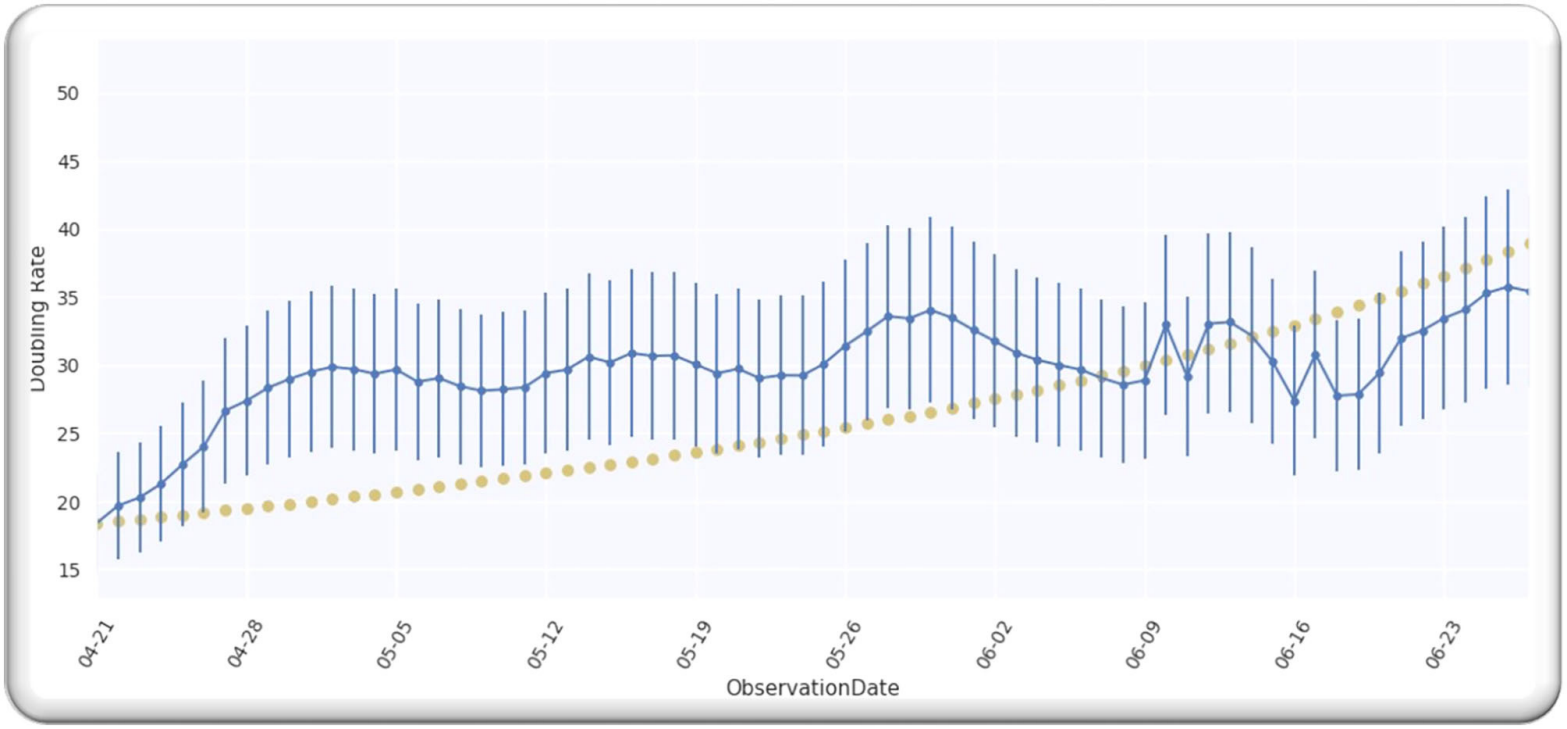
The doubling rate of cases in Asia with estimates.

Ratio $Y_{\min}^{2}/\left(M-1\right)$ = 0.97 that signifies that model is performing well on the data because the ratio is smaller than Value 1. Figure 7 illustrates the analyzed doubling rates with modeled doubling rates for the Asian countries.

The value of *D* is equal to 127 when *p* = 0 on 21/04/2020, comparable to 21/04/2020 + D, which is 08/08/2020. Then, for Asian countries, the best fit Gaussian doubling time is given by,


(18) $$\begin{array}{r} C\left(D,p\right)=\frac{18.42}{1-p/D}=\frac{18.42}{1-p/127}=\frac{2339.34}{127-p} \end{array}$$

**Figure 8:**
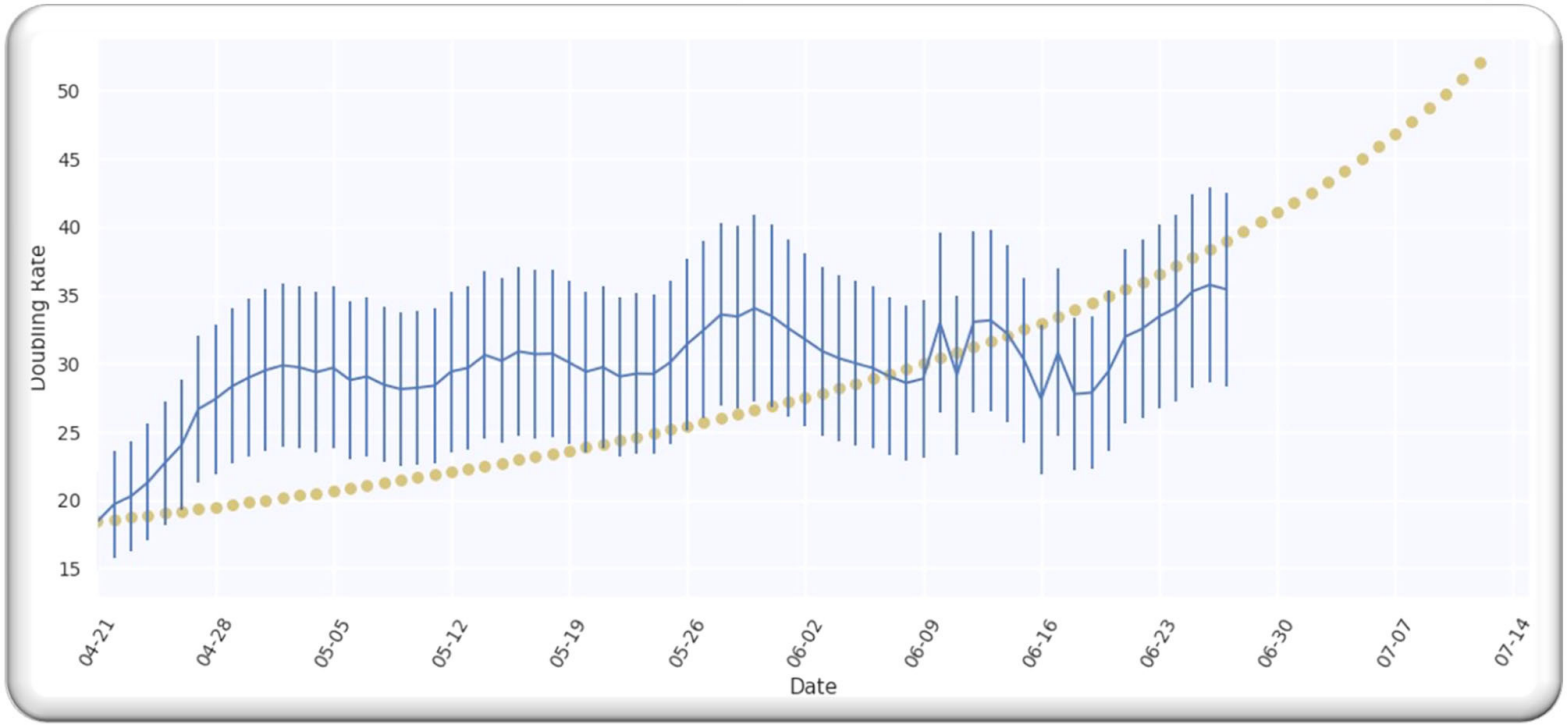
The doubling rate of cases in Asia with estimates.

The estimate of doubling rate up to 14/07/2020 for the Asian countries is illustrated in Figure 8. We have the value of *D*, which is equal to 127, so we can compute the Δ by using Equation (8).


(19) $$\begin{array}{r} 0.35\Delta^{2}=18.42D\Rightarrow \Delta =81.75 \end{array}$$

**Figure 9:**
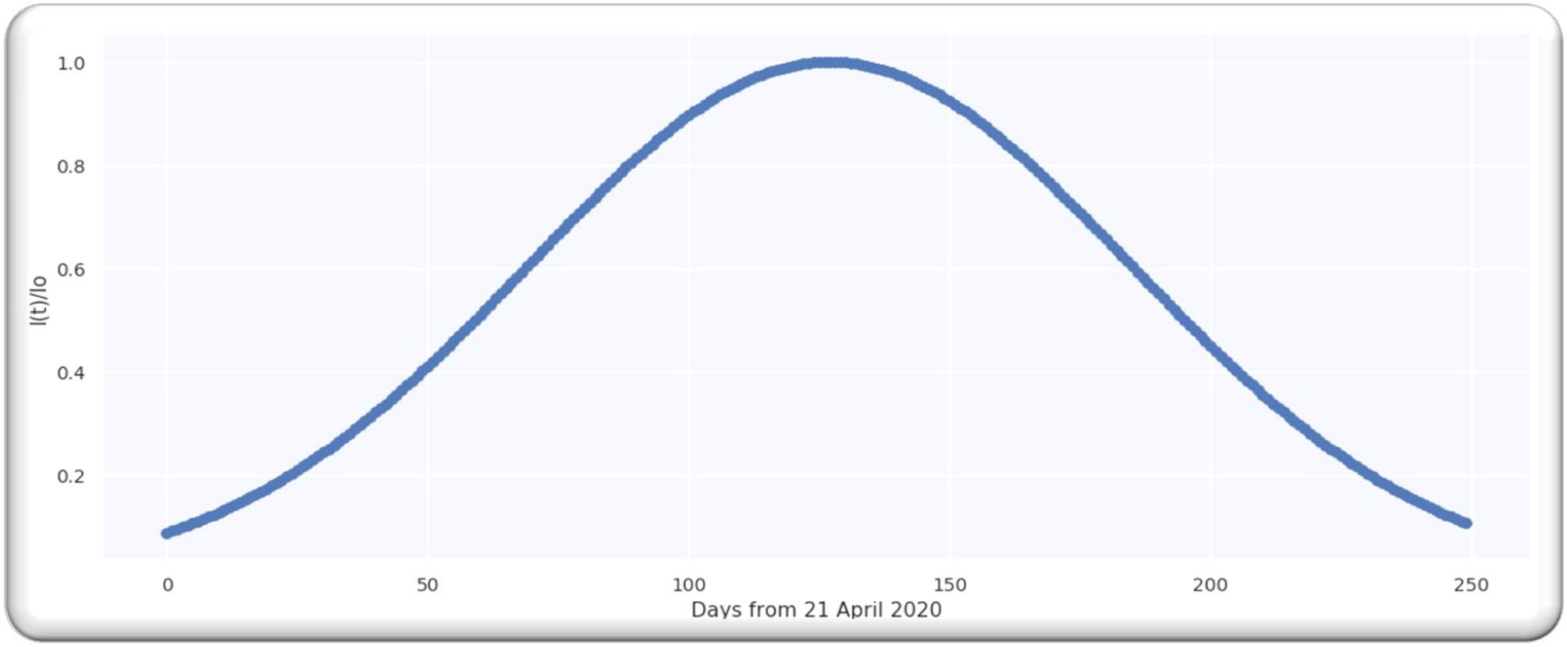
Gaussian function for cases in Asia.

For the Asian countries' infection cases, the Gaussian function in terms of the ratio of $\frac{k\left(p\right)}{k_{0}}$ is illustrated in Figure 9.

## Proposed Method

**Figure 10:**
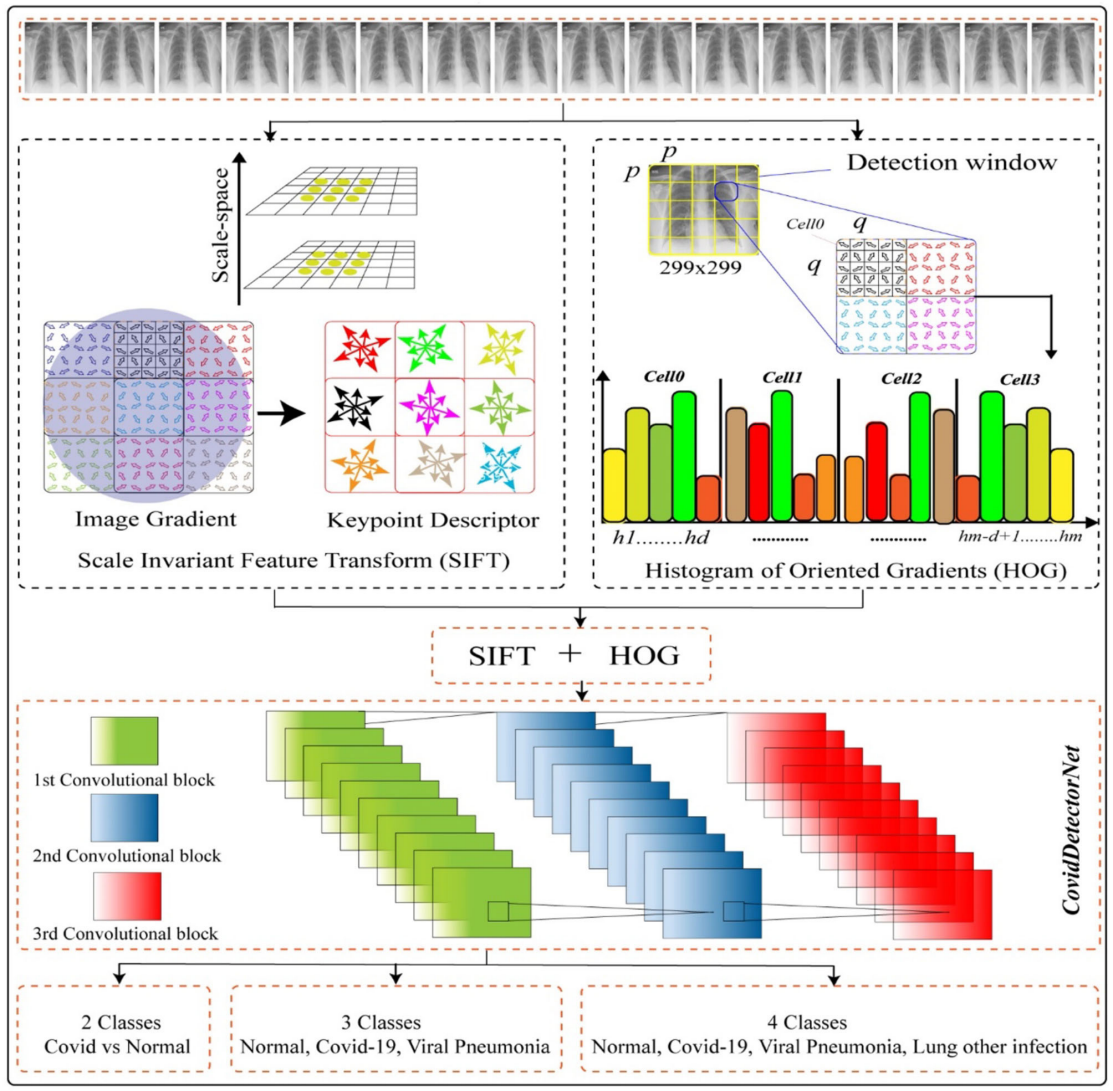
Proposed systems.

The major purpose of this working mechanism is to estimate the infection rate throughout the Asian countries as well as worldwide and to detect the COVID-19-infected people. The working mechanism comprises two stages, such as employing the two features, namely, histogram of oriented gradients (HOG) and the scale invariant feature transform (SIFT) on CXI and, next, passing the extracted features into the proposed CNN-based architecture named COVIDDetectorNet for further processing and prediction. The working of our method HOG-SIFT-COVIDDetectorNet is illustrated in Figure 10.

### COVID-19 Detection

#### Dataset

We employed a public dataset COVID-19 CHEST X-RAY DATABASE^1^ to perform all the experiments, such as two classes (COVID and normal), tree classes (COVID, normal, and VP), and four classes (COVID- normal, VP, and other lung infections). A team of researchers belonging to different countries, namely, the University of Doha, Dhaka, Qatar, Pakistan, and Malaysia, has collaboratively developed the dataset with the help of medical experts. The dataset has four different classes, where 3.616 CXI are of COVID; 6,012 of the other lung infections; 1,345 viral cases of pneumonia; and 10,192 of normal people. Each image has a resolution of 299 × 299 and a PNG extension.

### Features Extraction

#### Scale Invariant Feature Transform

In the initial stage, we employed the SIFT feature descriptor on the CXI to extract prominent features. SIFT captures the distinct characteristics based on the difference of a pixel gradient. Although the speeded-up robust features (47) have shown significant robustness as compared to the SIFT features, however, it has a high computational cost. The SIFT feature (48) was developed for extracting the unique invariant characteristics from the images, which can be utilized for performing dependable matching between the distinct views of a scene or an object. In order to extract SIFT features, we used a four-stage procedure, such as scale space and extreme detection, keypoint localization, orientation assignment, and keypoint descriptor. The detailed computation of the SIFT feature is as follows in the subsequent sections.

#### Scale-Space and Extreme Detection

In the initial phase of SIFT feature computation, we defined the scale space of the CXI as a function, F (a, b, σ), which is generated from the convolution of a variable-scale Gaussian kernel, *K* (a, b, σ) using the input CXI*I* (*a, b*).


(20) $$\begin{array}{r} F\;\left(a,b,\sigma \right)={K\left(a,b,\sigma \right)}^{*}I\left(a,b\right) \end{array}$$

where the symbol ^*^ shows the convolution operation in *a* and *b*, and


(21) $$\begin{array}{r} K\left(a,\;b,\;\sigma \right)=\frac{1}{{2\pi \;\sigma }^{2}}e^{\frac{-a^{2}+b^{2}}{{2\sigma }^{2}}} \end{array}$$

For the efficient detection of the stable keypoint locations in the scale space, we used the scale-space extrema in the difference of the Gaussian function, followed by the convolution of CXI as follows;


(22) $$\begin{array}{l} D\;\left(a,b,\sigma \right)={\left(K\left(a,b,\;k\sigma \right)-G\left(a,b,\sigma \right)\right)}^{*}I\left(a,b\right)\\ \;=F\left(a,b,k\sigma \right)-F\left(a,b,\sigma \right) \end{array}$$

The *k* in the Equation (22) shows the multiplicative factor.

For the detection of local maxima and minima of *D* (*a, b*, σ), we compared every sample point to its 8 neighbors in the CXI and the 9 neighbors in the scale upper and lower as shown in **Figure 13**.

#### Keypoint Localization

In the second stage of SIFT features computation, a candidate's key point that was detected in the initial stage is refined to a subpixel level, however, the unstable are eliminated. Moreover, non-edge points and noise-sensitive points are removed in keypoint localization for enhancing the stability of matching and enhancement of noise exemption. The extreme points of low contrast are eliminated by employing the Taylor series for expanding the scale-space function *D* (*a, b*, σ) at sampling point *S* (*a, b*, σ)^*T*^ (49). The trace and determinant ratios of the Hessian matrix are to decrease the edge effect of the difference of the gaussian function.

#### Orientation Assignment

In the third stage of the computation of SIFT features, the local information from the key points is extracted with identified location and scale. Based on the local characteristics of CXI, it decrypts the feature point location information, which makes the SIFT features remain unchangeable for the rotation of the image. An orientation histogram is produced by using the gradient orientations of neighboring pixels of keypoints. The keypoints can be assigned according to the histogram orientation as given in Equation (23).


(23) $$\begin{array}{l} m\;\left(a,b\right)\\ =\sqrt{{\left(F\left(a+1,b\right)-F(a-1,b)\right)}^{2}+{\left(F\left(a,b+1\right)-F(a,b-1)\right)}^{2}}\\ \theta \;\left(a,b\right) \end{array}$$

(24) $$={\text{tan}}^{-1}((F\left(a,b+1\right)-F\left(a,b-1\right))/(F\left(a+1,b\right)-F\left(a-1,b\right)))$$

The above two Equations (23) and (24) provide the modulus and the direction of the gradient at pixel (a, b); scale of the F is the corresponding scale of every keypoint. In actual computations, we achieved the neighborhood gradient direction through the statistics histogram and sample in the vicinity window centered at the keypoint. The range of gradient histogram is 0–360 degrees and 36 columns. There are a total of 36 bins in gradient histogram that cover 360 degrees of orientation. The dominant direction of the neighborhood gradient is shown by the peak of the histogram.

#### Keypoint Descriptor

**Figure 11:**
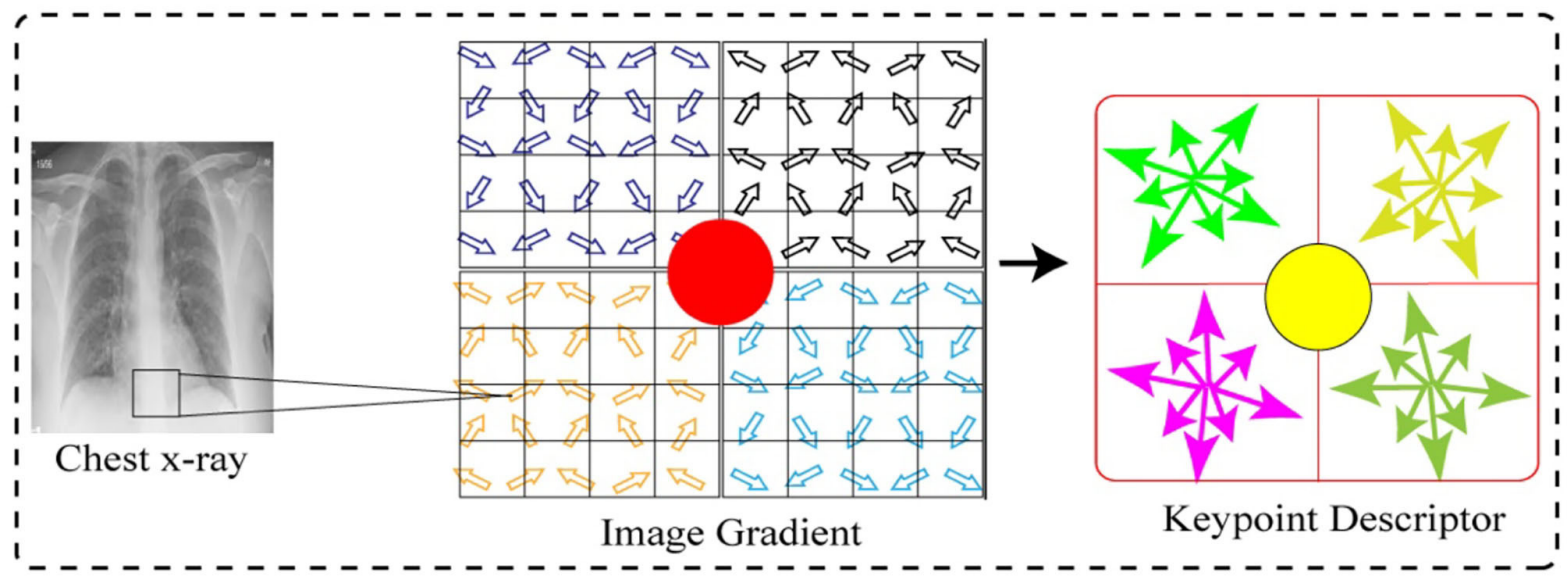
Keypoint descriptor and image gradient.

Figure 11 shows the feature point definition of the SIFT descriptor in a neighboring area, which maintains invariable to the angle of the view and brightness change. In order to make sure of the rotation invariance, the direction of axis is organized as the keypoint.

Next, we considered eight by eight windows around each keypoint. The central red highlighted part in Figure 11 is a current keypoint position. Every cell depicts a neighboring pixel in scale of the keypoint, while the arrow in each cell shows the gradient direction of each pixel. Moreover, the length of each arrow illustrates the mold value of the gradient, and the yellow circle illustrates the Gauss-weighting scope. The number of pixels close to the keypoint signifies the maximum contribution to gradient direction information.

Next, in each four by four sub-window, the gradient direction histogram is produced, having eight orientation bins, and it is called a seed point as illustrated in Figure 11. One keypoint has total two by two seed points as illustrated in Figure 11, each seed point has eight different pieces of vector information. This neighboring joint orientation information improves the capability of anti-noise algorithm.

### Histogram of Oriented Gradients

**Figure 12:**
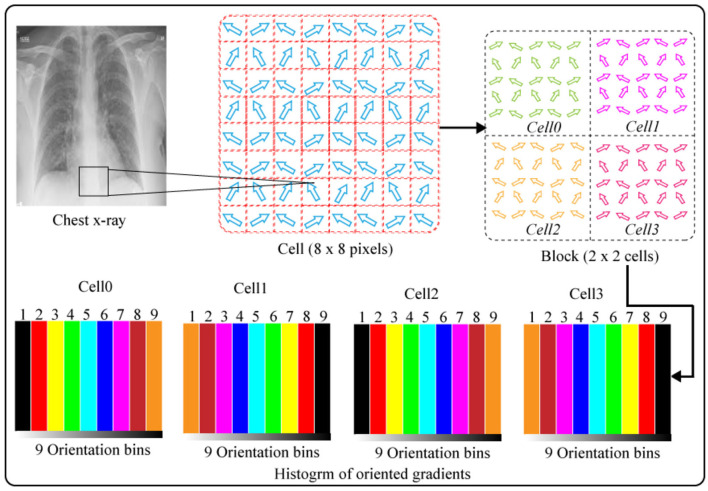
Histogram of oriented gradients.

HOG was originally developed (see footnote^1^) for characterizing images on gradient directions. This feature descriptor is employed in digital image processing as well as computer vision for classification and object detection. The major goal of the HOG algorithm is to analyze the histogram of an oriented gradient in areas of the neighboring images. Figure 12 illustrates the computation of HOG features. A given image is split up in numerous minors, as well as correlated zones named units. These units are again split up in groups of cells and different gradient directions. In this work, we extracted HOG features from the CXI as follows: in the initial stage, we split up an image portion of a sample having pixel size of (48 × 48) into minor cells of the same pixel size (8 × 8) and then calculated gradient histogram of each pixel in all cells through splitting up orientation into nine bins. The computation of nine-bin histogram for each cell creates an illustration significantly well and dense to an interference. Moreover, gradient sections of the CXI are calculated by employing the one-dimension balanced method in two different directions, namely, vertical and horizontal. Gradient sections are calculated by using the following Equations (25) and (26).

Gradient sections of CXI are calculated through the one-dimension centered method in horizontal and vertical directions. Gradient sections are calculated by employing the below Equations.


(25) $$\begin{array}{r} G_{a}\left(a,b\right)=P\;\left(a+1,b\right)-P\left(a-1,b\right) \end{array}$$

(26) $$\begin{array}{r} G_{b}\left(a,b\right)=P\;\left(a,b+1\right)-P\left(a,b-1\right) \end{array}$$

The *P* (*a, b*) indicates the pixel value, while the *G*_*a*_ (*a, b*), as well as *G*_*b*_(*a, b*), shows gradients in two directions of the pixels, i.e., horizontal and vertical, respectively. Moreover, we also calculated the magnitude and direction of the gradient, such as *z* (*a, b*) for each pixel (*a, b*) as below:


(27) $$\begin{array}{l} G\;\left(a,b\right)=\sqrt{G_{a}{\left(a,b\right)}^{2}+G_{b}{\left(a,b\right)}^{2}}\\ z\;\left(a,b\right) \end{array}$$

(28) $$\begin{array}{r} =\tan^{-1}\left(\frac{G_{b}\left(a,\;b\right)}{G_{a}\left(a,\;b\right)}\right) \end{array}$$

The orientation for gradients ranges from 1–18^0^. We calculated both the orientation and magnitude for every pixel in each cell. Finally, we normalized the histogram for every cell and grouped the histograms of all the cells to illustrate a single block. The histograms block depicts the HOG features of the CXI. HOG features have advantage of preserving the spatial characteristics of images.

### Convolutional Neural Networks

DL has a sub-branch called an artificial neural network that is inspired by the living organisms' natural visual perception working (50). The CNNs are multi-layered neural networks (NNs) stacked together that comprise mainly three types of layers, such as convolutional layers, pooling layers, and fully connected layers. The very first layer of each CNN model is an input layer; the depth, height, and width of input images are specified as input parameters. Instantly, after the initial layer, some stacked convolutional layers are defined with different configurations and parameters, such as hidden unit size, number of filters, padding, stride, and activation functions. The convolutional layers are responsible to extract significant feature maps from the inputs by computing the weighted sum (51, 52). The extracted feature maps are then passed through the activation functions, and a bias is added to obtain an output. Typically, the rectilinear unit (ReLU) is employed as an activation function (53). Moreover, the pooling layers are employed for reducing the size of the output from the lagging convolutional layers. The output dimensionality increases exponentially with the increase in the size of the model by increasing the number of input parameters to the convolutional layers, which is challenging for low computational cost machines. To avoid the above problem, pooling layers are employed to minimize the dimensions for simple and easy computation. The pooling layers are also used to suppress the noise as well. There are numerous pooling layers, such as max, avg, global, and spatial pooling layers; however, the researchers employ the max-pooling layer (54). Output is flattened to produce a single-array feature vector, which is then passed into the fully connected layer. Finally, a dense layer referred to as the classification layer is defined as having an activation function, such as SoftMax, tanh, sigmoid, etc. (55). The number of classes used for experimentation purposes is specified in the last layer, and the feature maps are combined into class scores. In the CNNs, there are batch normalization layers that are employed after the initial layer or just after an activation layer for standardizing the learning process and minimizing the time of training (56). Moreover, a significant parameter is a loss function that summarizes the error during the training time and validation time in the predictions. The loss of the model is backpropagated into the CNNs after every epoch to optimize the process of learning (57).

#### Proposed COVIDDetectorNet Architecture

**Figure 13:**
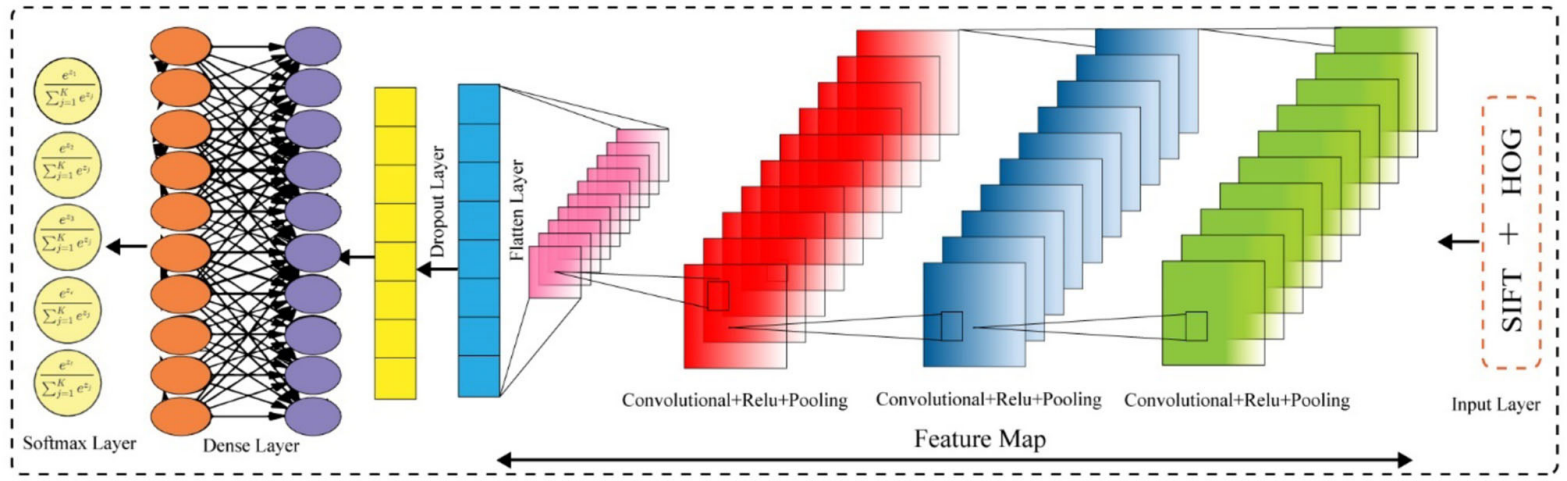
Proposed COVIDDetectorNet.

In this work, we proposed a CNN-based architecture, namely, COVIDDetectorNet. The research community employs CNN-based architectures for image analysis due to the improved performance in the image processing field. The convolutional layers and numerous filters, i.e., 3 × 3, 5 × 5, 7 × 7, 9 × 9, 11 × 11, etc., assist to extract both the spatial and temporal features from images. The convolutional layers comprise the weight-sharing technique, which assists to reduce the computational costs (58, 59). We fed the fused features, such as HOG and SIFT, into the COVIDDetectorNet for classification purposes. The proposed COVIDDetectorNet consists of three sections, namely, blocks of convolutional layers, followed by the max-pooling layers, and, finally, a dense layer, followed by a SoftMax layer. The initial layers are utilized for extracting features from CXI; max-pooling layers are employed for sub-sampling purposes, which down-sample images and reduce the dimension, so they minimize the computation costs and efforts, while the dense layer classifies the images. Our COVIDDetectorNet architecture has three convolutional layers, and we employed a max-pooling layer after each convolutional layer. The first two convolutional layers used kernels of 5 × 5, and the last convolutional layer used a kernel of 3 × 3. Moreover, we employed three max-pooling layers, and all have the same sizes of 2 × 2 as well as a dropout layer of fives for reducing the overfitting problem. After all, we utilized the dense layer, followed by a SoftMax layer to classify the two classes (COVID and normal), tree classes (COVID, normal, and VP), and four classes (COVID- normal, VP, and other lungs infections). The architecture of the proposed COVIDDetectorNet is illustrated in Figure 13.

## Results and Analysis

In this portion, we discussed the experimental outcomes as well as the explanations of numerous experiments performed for measuring the performance of the proposed system.

### Detection Performance of COVID-19

We developed a multiphases experiment for the detection of patients with COVID using CXI by employing the HOG and SIFT features and fed the extracted features into the proposed COVIDDetectorNet for classification. This experiment comprises three stages, namely, (detection of normal vs. COVID), (normal, COVID, and VP), and, finally, (normal, COVID, VP, and extra lungs infections).

In the very first phase of this experiment, we evaluated the performance of our method using two classes, namely, normal and COVID for the detection of COVID-infected persons. To achieve this goal, we utilized 10,192 and 3,616 CXI of normal and COVID-19 infected persons for the detection of COVID-19 individuals. Primarily, we split up all the CXI into 90 by 10, whereas, we used the 90% (12,431 CXI) to train the COVIDDetectorNet and 10% (1,377 CXI) to test the trained COVIDDetectorNet. Next, we employed HOG and SIFT to extract prominent characteristics from CXI. Finally, we fed the extracted HOG and SIFT features of both classes, i.e., normal and patients with COVID-19 into the proposed COVIDDetectorNet. The Table 1 shows the detailed results for the two classes. We obtained an accuracy, precision, recall, F1-score of 96.51, 97.67, 97.73, and 97.20%, respectively. The above experimental results show that our method (HOG-SIFT-COVIDDetectorNet) performs exceptionally well to detect the COVID-19-infected people and can be employed in real-time environments because the precision rate of our method is greater than the standard PT-PCR tests.

**Table 1:** Detection performance of COVID-19 on 2, 3, and 4 classes.

**No of classes**	**Accuracy %**	**Precision %**	**Recall %**	***F*1-score %**
2	96.51	97.67	97.73	97.20
3	92.62	91.09	91.95	91.52
4	86.53	87.19	86.34	86.22

Next, we evaluated the performance of our method (HOG-SIFT-COVIDDetectorNet) in the three-class scenario, namely, normal, COVID, and VP, to detect VP patients along with the patients with COVID-19. To accomplish this goal, we utilized 10,192 of normal, 1,345 of VP, and 3,616 CXI of COVID-19-infected people. Furthermore, we split up the data of three classes into 90/10 sizes and employed the 13,638 CXI for training the COVIDDetectorNet, while the 1,515 CXI for evaluating the trained COVIDDetectorNet. Again, we employed HOG and SIFT on CXI of three classes to extract the features. Next, the extracted features are fed into the proposed COVIDDetectorNet for detection purposes of normal, COVID-19, and VP patients. As illustrated in Table 1, our method obtained an accuracy, precision, recall, and F1-score of 92.62, 91.09, 91.95, and 91.52%, respectively, for the three classes. These results on the three classes show that fused sets of HOG and SIFT features have ability to preserve most crucial characteristics of an image and the proposed COVIDDetectorNet to effectively classify the normal, COVID-19, and VP patients.

In the final phase of this experiment, we evaluated the performance of HOG-SIFT-COVIDDetectorNet on four different classes, namely, normal, COVID-19-infected, VP patients, and other lung infections to demonstrate the robustness in the multi-class problem of our method. To achieve this goal, we added 6,012 x-ray images into the data of three classes (normal, COVID, and VP) that were utilized in the second phase of this experimentation. We again split up the data into 90% (19,039 x-ray images) and 10% (2,128 x-ray images). Moreover, we utilized 90% for training and 10% for evaluating the COVIDDetectorNet. We extracted the HOG and SIFT features from x-ray images of all the four classes and fed the fused set of features into the proposed COVIDDetectorNet for classification purposes. Table 1 illustrates the detailed results of our technique in a multi-class scenario. More specifically, we obtained an accuracy, precision, recall, and F1-score of 86.41, 87.19, 86.34s, and 86.22%, respectively. These experimental outcomes reveal that our technique is capable to accurately detect all the four classes.

### Error Matrix Analysis

We developed an error matrix for representing the classification evaluation of our technique to determine the accurate and wrong prediction for all the four classes. Keeping in mind the fact that error matrix shows the performance of each class, we also developed three error matrices for three different experiments, namely, two classes, three classes, and four classes for advanced visualization of our technique.

In the first phase, we developed an error matrix to visualize performance of HOG-SIFT-COVIDDetectorNet on two classes, namely, normal and COVID, as illustrated in Table 2. We can examine from the Table 2 that our technique correctly detected 1,994 and 622 x-ray images as normal and COVID, respectively, while 64 and 74 x-ray images of normal as COVID and COVID as normal, respectively. The FP and FN rates are 3.37 and 3.85%, respectively. These lower FP and FN rates signify that our technique is much dependable than the standard PT-PCR tests because the precision rate of PT-PCR is about 80–85%.

**Table 2:** Error matrix for two classes.

**Predicted class**	**Actual class**		
		**Normal**	**Covid-19**
	Normal	1007	24
	Covid-19	34	622

Next, we developed an error matrix for visualizing the performance of our technique in a multi-class scenario, i.e., for three classes, namely, normal, COVID, and VP, as illustrated in Table 3. As illustrated in Table 3, we can examine that our technique correctly detected 1,003, 283, and 122 x-ray images of normal, COVID, and VP, respectively. Moreover, our technique also incorrectly detected 128 x-ray images. The detailed classification results of each class are given in Table 3. The FP and FN rates are 2.67 and 4.93%, respectively. The experimental outcomes clearly signify the superiority of our technique to detect the presence of VP and COVID in people.

**Table 3:** Error matrix for three classes.

**Predicted class**	**Actual class**			
		**Normal**	**Covid-19**	**VP**
	Normal	1003	31	5
	Covid-19	61	283	2
	VP	2	7	132

In the final phase, we developed an error matrix for visualizing the performance of our technique on the four classes to detect COVID-infected people in a multi-class environment, as illustrated in Table 4. Table 4 reveals that our technique has correctly detected 478, 933, 319, and 119 x-ray images of normal, COVID, VP, and lung infection, respectively. The proposed method also incorrectly detected 279 x-ray images. The details of correct and incorrect classification for each class are given in Table 4.

**Table 4:** Error matrix for four classes.

**Predicted class**	**Actual class**				
		**Normal**	**Covid-19**	**VP**	**Lung infection**
	Normal	478	85	35	3
	Covid-19	59	933	27	9
	VP	15	30	319	0
	Lung infection	5	8	3	119

## Discussion and Conclusion

The life-threatening novel COVID-19 has spread to more than 224 countries, and, by the end of February 2022, 439 million people are infected, and 5.96 million deaths are reported from all over the world; however, some counties, namely, USA, Asia, Europe, etc., are severely affected by this fatal virus. This work focuses on designing, examination, and the simulation of a novel robust technique to facilitate the in-depth assessment of quick spread as well as the prevention of COVID globally. The proposed technique comprises two tasks, such as infection forecasting and COVID detection. For the COVID forecasting, we proposed a Gaussian model that assists in unassuming predictions of COVID infections. Moreover, we developed a very first indication that the proposed forecasting model is smart to capture the daily time evolution of fatalities as well as the infections rate for each country. Appropriate simulations present the past data and the data of China. Our developed Gaussian model is very flexible, which can be simulated and performed short of prior information of epidemiologic, data, or any programs. Still, there are countries that are not badly exaggerated by the pandemic and will change in next coming weeks. Hence, our Gaussian model can be employed in the countries that are not badly affected as soon as enough data become available for forecasting. The monitoring authority of COVID can obtain forecast for the shape of the Gaussian curve for their own countries by employing the Gaussian model. Moreover, the public bodies, as well as the governments, can employ our forecasting model to calculate additional measures of interest, i.e., forecasting the maximum possible number of machines used for respiratory diseases and the deadline for the maximum requirement. The total amount and circulation of SSPs can let the health agencies and COVID administrative authority in countries to improve the administration of pandemic waves by taking drastic, effective, and time-limited measures. Furthermore, fortunately, our assessment signifies here that the peak time of each wave significantly varies from country to country. To predict the peak times and relevant time frames assists other countries to make an advantage from those who has witnessed the peak of the wave, expectable duration with respiratory diseases equipment, and medical experts at a marginally delayed time. In the COVID-detection framework, we employed two feature descriptors, such as HOG and SIFT, from the CXI. Moreover, we also designed a CNN-based architecture, namely, COVIDDetectorNet for the classification of two, three, and four classes. Our method has shown remarkable performance to detect COVID-infected people in binary classification, ternary classification, and quaternary classification problems. The remarkable results of all the classes show that HOG-SIFT-COVIDDetectorNet has performed exceptionally well, and this can be employed in emergency services, hospitals, airports for screening, and any other organizations for screening patients with COVID-19. In the near future, our aim is to perform experimentation on other variants as the data become available.

## Data Availability Statement

The original contributions presented in the study are included in the article/supplementary material, further inquiries can be directed to the corresponding author/s.

## Author Contributions

FH: conceptualization, formal analysis, and data analysis. SA: data interpretation, literature search, funding acquisition, conceptualization, software, resources, methodology, and writing-original draft. AJ: supervision, validation, and writing-review and editing. AI: conceptualization, supervision, writing-review and editing, and proofreading. All authors listed have made a substantial, direct, and intellectual contribution to the work and approved it for publication.

## Conflict of Interest

The authors declare that the research was conducted in the absence of any commercial or financial relationships that could be construed as a potential conflict of interest.

## Publisher's Note

All claims expressed in this article are solely those of the authors and do not necessarily represent those of their affiliated organizations, or those of the publisher, the editors and the reviewers. Any product that may be evaluated in this article, or claim that may be made by its manufacturer, is not guaranteed or endorsed by the publisher.

## Footnotes

^1^Available online at: http://www.chest-x-rays-radiographicimages.com.
